# Relative quantification of TCR *Vbeta*-chain families by real time PCR for identification of clonal T-cell populations

**DOI:** 10.1186/1479-5876-6-34

**Published:** 2008-07-01

**Authors:** Sebastian Ochsenreither, Alberto Fusi, Antonia Busse, Dirk Nagorsen, David Schrama, Jürgen Becker, Eckhard Thiel, Ulrich Keilholz

**Affiliations:** 1 University Hospital Benjamin Franklin, Medizinische Klinik III, Hematology, Oncology, and Transfusion Medicine, Charité Universitätsmedizin Berlin, 12200, Berlin, Germany; 2 University of Würzburg, Clinic for Dermatology, Allergology, and Venerology, 97080, Würzburg, Germany

## Abstract

**Background:**

Quantification of T-cell receptor (TCR) chain families can be utilized for detection of clonal T-cell populations. Besides southern blotting and antibody-based approaches, quantitative real time PCR (qRT PCR) has been more widely applied in this context during the last years. Here, the heterogeneity of sequences within single families is the most challenging problem for exact quantification.

**Method:**

*Vβ*-families were quantified using a universal reverse primer and family-specific forward primers with TaqMan technology on a light cycler instrument. Relative concentrations were calculated considering slopes and crossing points of each PCR reaction. Total expression of *α*/*β *TCR was assessed by quantification of the constant *α*-chain as a further control.

**Results:**

The method was tested by serial dilutions of clonal T-cells in mononuclear cells from healthy volunteers. Calculated percentages were in good correspondence with qRT PCR results demonstrating high reliability. Duplicates showed excellent technical reproducibility. We analyzed blood samples of 20 healthy volunteers for determination of mean and standard deviation for each family. The method was applied both to tissue and blood samples from patients with carcinomas and hematological disorders.

**Conclusion:**

We introduce a versatile method for the relative quantification of *Vβ*-families by real time PCR. The experimental strategy described allows the identification of alterations in the *Vβ*-family repertoire.

## Background

T-lymphocytes are specialized mediators of the adaptive immune system, selectively destroying cells altered by viral infection or malignant transformation [1,2]. T-cell mediated immune responses are characterized by activation and subsequent clonal expansion of antigen-specific cells. Recognition of Major Histocompatibility Complex class I (MHC-I) bound peptide is mediated by the dimeric transmembrane T-cell receptor (TCR) composed of an *α*- and a *β*-chain in the majority of cases. The high diversity of these chains is generated by stepwise recombinations of a multitude of variable (*V*), in case of the *β*-chain diversity (*D*), and joining (*J*) gene sequences with a corresponding constant (*C*) chain during thymic T-cell evolution [3]. *V*-genes are grouped in families consisting of genes with at least 50% sequence homology [4].

T lymphocyte repertoire alterations can be evaluated by TCR-diversity restriction analyses of the sequence or the length of the high variable part of the *α*- or *β*-chains (e. g. spectrotype, SSCP ('single strand conformation polymorphism'), DGGE ('denaturing gradient gel electrophoresis'), heteroduplex analysis [5-11]) or by TCR *V*-family quantification [12-16]. Because of the lower described number of *Vβ*-families compared to *Vα*-families, the higher variability of the *β*-chain, and the fact that each T-cell clone can express two different *α*-chains but only one *β*-chain, the *Vβ*-chain has been largely preferred for this type of analysis [12,13,17,18].

Quantification of *Vβ*-families have been investigated by means of different approaches as southern blotting or, more recently, Fluorescence Activated Cell Sorter (FACS) and quantitative real time reverse transcribed PCR (qRT PCR) [5,12-14]. In contrast to other approaches, PCR-based methods could detect a higher number of different families, had higher selectivity, and were applicable to different specimens. A cumbersome limitation for qRT PCR assessments was the suboptimal establishment of standard dilution curves for exact quantification due to the high heterogeneity of sequences within each family.

Here we introduce a versatile and rapid method for qRT PCR relative quantification of *Vβ*-family expressions based on slope and crossing point of the respective PCR reaction overwhelming therefore the problem of establishing standard dilution curves.

## Methods

### Specimen collection

Peripheral blood samples were drawn form patients and healthy volunteers. Tissue samples were collected from patients with colorectal cancer who underwent therapeutical resection. Both patients and controls had given informed consent for the use of their specimens before sampling. Human T acute lymphoblastic leukemia (ALL) cell lines JURKAT, MOLT-16, and CCRF-CEM were purchased from German Collection of Microorganisms and Cell Cultures (DSMZ) and cultivated under recommended conditions.

### RNA extraction and cDNA synthesis

Total RNA was extracted from peripheral blood mononuclear cells (PBMCs) or fresh tissue using TRIzol^® ^(Invitrogen, Carlsbad, California, USA) or RNeasy^® ^Mini Kit (Qiagen, Hilden, Nordrhein-Westfalen, Germany) according to manufacturers' instructions. RNA was quantified using a NanoDrop^® ^ND-1000 spectrophotometer (Thermo Fisher Scientific, Wilmington, Delaware, USA), and integrity was checked electrophoretically. Reverse transcription was performed with Omniscript Reverse Transcriptase^® ^(Qiagen) as described previously [19]. Samples were stored at -20°C.

### Relative quantification of Vβ-families

**Figure 1:**
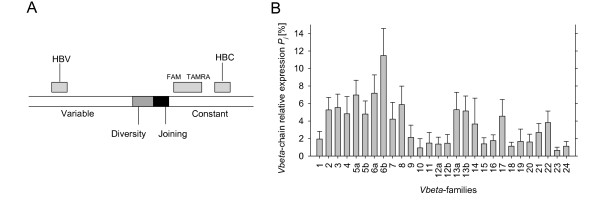
**Primers and probe annealing sites and Vβ-families distribution in peripheral blood of healthy volunteers**. (A) Structure of the *β*-chain and annealing sites of primers and of the TaqMan probe. Forward primers (HBV) anneal at the variable region, reverse primer (HBC) and TaqMan probe anneal at the constant region (B) Average percentages and standard deviations for *Vβ*-families in PBMCs obtained from 20 healthy controls. Family percentages *Pj *[%] reflecting single family expression by the sum of all measured *Vβ*-families are shown.

For estimation of the relative expression of a single TCR *Vβ*-chain, qRT PCRs were performed with a universal reverse primer HBC-rev, a TaqMan probe HBCTP-FAM, both annealing at the constant part of the *β*-chain (*Cβ*), and *Vβ*-family-specific forward primers (modified from [10], Table 1, Figure 1A). Nomenclature of *Vβ*-primers corresponded to the classification of Arden et al [4]. A Mastermix of 19 μl for each of the 29 reactions (LightCycler TaqMan Master^®^, Roche, Basel, Switzerland) containing 0.5 μM reverse primer HBC-rev, 0.1 μM TaqMan-probe HBCTP-FAM, and 2 μl template cDNA was prepared. For *Vβ*-family specific amplification, the corresponding forward primer was added to a final concentration of 0.5 μM. A negative control without forward primer was added. Amplification was performed after an initial denaturation step of 95°C for 1 min in 45 cycles of 95°C for 10 sec, 60°C for 30 sec, and 72°C for 1 sec with data aquisition. The cycling was followed by a terminal elongation step at 72°C for 2 min. Crossing points were determined using the Fitpoint algorithm as implemented in LightCycler software 3.0. Slope of each family specific reaction was determined by linear regression analyzing a dilution series (1, 0.1, and 0.01) of a cDNA mixture of diagnostic samples. The average of these slopes $\bar{s}$ was used for determination of *c' *as described below.

**Table 1:** Primers/probe for quantification of *Vβ*-families (modified from [10], TaqMan).

Primer	Sequence (5' – 3')
HBV1.1	CAC TCT GAA CTA AAC CTG A
HBV2	TCA ACC ATG CAA GCC TGA CC
HBV3	CGC TTC TCC CTG ATT CTG GAG TCC
HBV4	TTC CCA TCA GCC GCC CAA ACC TA
HBV5A	CTG AGA TGA ATG TGA GCA CCT TG
HBV5B	CTG AGC TGA ATG TGA ACG CCT TG
HBV6A	AGA TCC AGC GCA CAG AGC G
HBV6B	AGA TCC AGC GCA CAS AGC A
HBV7	GCC AAG TCG CTT CTC ACC TG
HBV8	TGA AGA TCC AGC CCT CAG AAC CC
HBV9	TCT CAC CTA AAT CTC CAG ACA AAG
HBV10	CCA CGG AGT CAG GGG ACA CA
HBV11	TGC CAG GCC CTC ACA TAC CTC TCA
HBV12A	GAG AAT TTC CTC CTC ACT CTG G
HBV12B	GAC CTC CCC CTC ACT CTG G
HBV13A	CTC AGG CTG CTG TCG GCT G
HBV13B	CTC AGG CTG GAG TTG GCT G
HBV14	AGG GTA CAA AGT CTC TCG AAA AG
HBV15	CAG GCA CAG GCT AAA TTC TCC
HBV16	GAA CTG GAG GAT TCT GGA GT
HBV17	GAA GGG TAC AGC GTC TCT CGG
HBV18	TTT CTG CTG AAT TTC CCA AAG AGG
HBV19	TCT CAA TGC CCC AAG AAC GCA C
HBV20	AGG TGC CCC AGA ATC TCT CAG
HBV21	TCA AAG GAG TAG ACT CCA CTC TC
HBV22	AGA TCC GGT CCA CAA AGC TG
HBV23	ATT CTG AAC TGA ACA TGA GCT CCT
HBV24	ATC CAG GAG GCC GAA CAC TTC
HBCTP-FAM	FAM-ATG GCT CAA ACA CAG CGA CCT CGG-TAMRA
HBC-rev	GGT GTG GGA GAT CTC TGC TTC

FAM: 6-carboxyfluorescin; TAMRA: 6-carboxytetramethylrhodamine, S: C or G

For the calculation of the percentage *P *of each *Vβ*-family *j*, it was assumed that the effective concentration *c *of each marker was

$$c=10^{\left(a+\frac{C_{p}}{\bar{s}}\right)}=10^{a}\cdot 10^{\frac{C_{p}}{\bar{s}}}$$

where *C*_*p *_was the Crossing point, $\bar{s}$[Δ*C*_*p*_/*log*(*c*)] was the mean slope, and *a *was considered as an unknown constant for normalization. Defining a linear concentration equivalent $c'=10^{\left(\frac{C_{p}}{\bar{s}}\right)}$, the percentage *P *[%] of a *Vβ*-family *j *equaled

$$P_{j}=\frac{10^{a}\cdot c{'}_{j}\cdot 100}{\sum_{i=1}^{n}\left(10^{a}\cdot c{'}_{i}\right)}=\frac{c{'}_{j}\cdot 100}{\sum_{i=1}^{n}c{'}_{i}}$$

assuming a constant *s *for all families.

### Quantification of TCR constant α-chain

For determination of total TCR expression, the TCR constant *α*-chain (HAC) was quantified on a LightCycler^® ^(Roche) as well as the low-abundance housekeeping gene porphobilinogen deaminidase (PBGD) for normalization as described [19]. PCR reactions were optimized using a mixture of cDNA as template. For quantification, LightCycler^® ^FastStart DNA Master HybProbe (Roche) was used. Primer sequences, final MgCl_2 _concentration, and annealing temperatures *T*_*A *_were listed in Table 2. After optimization of PCR conditions, fragments were amplified conventionally, cloned into a pCR2.1-TOPO vector, and transformed into TOPO10' competent *E. coli *using TOPO TA Cloning^® ^Kit (Invitrogen). After subcloning in liquid ImMedia^® ^(Invitrogen) and plasmid preparation with QiaMiniprep^® ^Kit (Qiagen), clones were linearized by digesting with *EcoR I *(Fermentas, St. Leon-Rot, Baden-Württemberg, Germany). Clones were sequenced, quantified spectrophotometrically and serially diluted as concentration standards. Sample concentrations were extrapolated plotting sample-crossing point *C*_*p *_against the regression line through standard-*C*_*p*_. RNA quantity was expressed as ratio plasmid equivalent HAC to PBGD.

**Table 2:** Primers/probes sets for quantification of PBGD and *Cα*-chain (FRET).

Primer/probe	Sequence (5'-3')	T_A_	MgCl2 (mM)	Standard dilutions (pg/μl)
PBGD-fwd	TGC AGG CTA CCA TCC ATG TCC CTG C	65°C	4	1/0.1/0.001
PBGD rev	AGC TGC CGT GCA ACA TCC AGG ATG T			
PBGD-3Fl	CGT GGA ATG TTA CGA GCA GCA GTG ATG CCT ACC-Fl			
PBGD-5LC	LC-TGT GGG TCA TCC TCA GGG CCA TCT TC-ph			
HAC2-fwd	ACA CCT TCT TCC CCA G	60°C	3	10/1/0.01
HAC2-rev	TCC AGT TGG TGG CAT T			
HAC2-3Fl	GTG ATT GGG TTC CGA ATC CTC C-Fl			
HAC2-5LC	LC-CTG AAA GTG GCC GGG TTT AAT CT-ph			

T_A_: Annealing temparature; Fl: Fluorescine; LC: LC Red 640; ph: phosphate

## Results and Discussion

Slopes of the PCR reactions for all *Vβ*-families were determined by dilution series of a positive control as described (data not shown). The average slope was 3.966 with a standard deviation of 0.61. This value was used for the calculation of family percentages. For assessment of mean value and standard deviation of each family in peripheral blood, PBMCs of 20 healthy volunteers were analyzed (Figure 1B).

**Figure 2:**
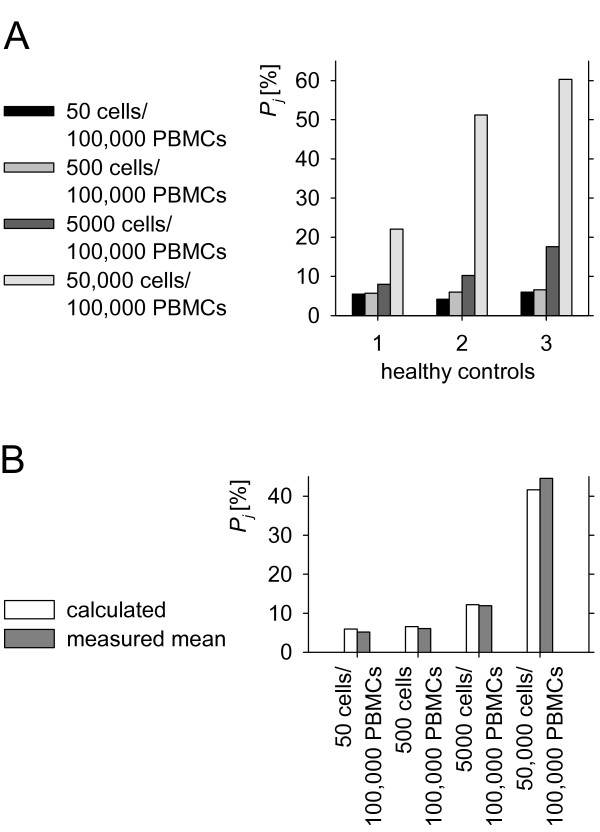
**Serial dilutions of JURKAT cells expressing Vβ-family 8 spiked in PBMCs of three healthy volunteers**. (A) measured percentages of family 8 in case of 50, 500, 5000, 50 000 JURKAT cells added to 100 000 PBMCs; (B) mean percentages of the three volunteers compared with theoretical values calculated assuming 5.88% family 8 without JURKAT cells and 70% TCR α/*β *positive cells in 100 000 PBMCs. *P*_*j*_: *Vβ*-chain relative expression.

**Figure 3:**
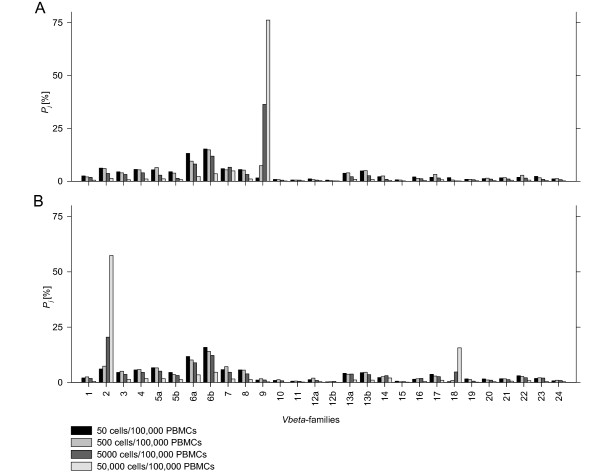
**Serial dilutions of CCRF-CEM (Vβ9) and MOLT-16 (Vβ2, Vβ18) cell lines in PBMCs of a healthy volunteer**. Measured percentages of all families in case of 50, 500, 5000, 50 000 CCRF-CEM cells (A) or MOLT-16 cells (B) added to 100 000 PBMCs of volunteer 2. Dilutions, RNA extraction, cDNA synthesis and qRT PCR are performed in duplicated. Mean percentages of each dilution step were shown. *P*_*j*_: *Vβ*-chain relative expression.

For assessment of reliability and reproducibility, samples containing 10^5 ^PBMCs of three healthy controls were prepared and different amounts of JURKAT-cells were added. JURKAT is a TCR *α/β*-expressing T-ALL cell line. Using serial dilution, aliquots of 50, 500, 5000, and 50,000 JURKAT cells were produced and subsequently added to PBMCs. Sample preparation, RNA extraction, cDNA synthesis, and qRT PCR were performed in duplicates for all concentrations for each volunteer. Results were expressed as averages of the calculated percentages of the sample duplicates. The mean difference in the percentage of each *Vβ*-family in all dilution duplicates was 1.01% indicating excellent technical reproducibility. For all three healthy control PBMCs spiked with JURKAT cells, a clear increase in percentage value of *Vβ*-family 8 was observed comparing 5000 to 500 cells per 10^5 ^PBMC (Figure 2A). Assuming 70% of mononuclear cells in peripheral blood expressing *β*-chains and 5.88% of the tested PBMCs belonging to *Vβ8 *(average percentage from 20 healthy controls), we calculated the theoretical percentages of family 8 dependent of the amounts of JURKAT cells added, which were in good correspondence with the mean of the measured values from the three healthy controls as shown in Figure 2B. As further validation, cell line MOLT-16 and CCRF-CEM were spiked in PBMCs of volunteer 2 as described above (Figure 3). Percentages of families not expressed by the cell lines relatively decreased at higher amounts of spiked cells. MOLT-16 weakly expressed a second *Vβ*-family, which became apparent at 5000 MOLT-16 cells per 10^5 ^PBMCs.

Because total amount of TCR α/β expression could be different among various types of specimens, we quantified the constant α-chain as a control of sample quality. HAC expression was 50-fold higher in PBMCs than in tissue samples (data not shown).

In this study, we introduced a new approach for estimating the distribution of *Vβ*-family expression in a population of T-cells. To determine the effective concentration of a transcript, standard dilutions of the specific fragment are generally utilized. In case of *Vβ*-family analysis, not a single target but a big amount of different close related transcripts has to be quantified because of the high variability of the Complement Determining Region 3 (CDR3) both in length and sequence. Therefore, establishment of a PCR in which a dilution series of a single amplicon (standard curve) is used for normalization, besides increase in costs, can not lead to exact results even using probes annealing to the constant part of the *Vβ*-chain. As PCR reactions lead to an exponential increase of produced fragments, and as a result crossing point values have to be interpreted logarithmically concerning the effective concentration of the target, it is possible to calculate percentages without normalization and independently from the sample concentration when crossing points and slope are taken into consideration. For these reasons, in order to by-pass CDR3 diversity, we established a system in which relative quantification is based on the comparison of crossing points, rather than performing a family-by-family quantification using single sequence plasmids for normalization.

There are two drawbacks on the approach: First, because the system is ,normalized' by the sum of its *c'*-values, results from runs with more than three lacking crossing points (because of suboptimal sample quality or technical problems) will not be informative. Second, the high standard deviation of the slopes limits the correctness of the mathematical procedure. Exemplary calculations showed that this effect was of no practical relevance as long as the concentration difference between compared samples, reflected by the difference between the sums of all *c' *(*c'*_*total*_), did not exceed a factor of 100 (data not shown). Nevertheless, this effect may play a role comparing blood with tissue as HAC/PBGD ratio in tissue is lower than in PBMCs. If the difference would be much higher, mathematical corrections of the percentage of a single predominant family should be performed considering *Δc'*_*total *_and *s*_*j*_.

**Figure 4:**
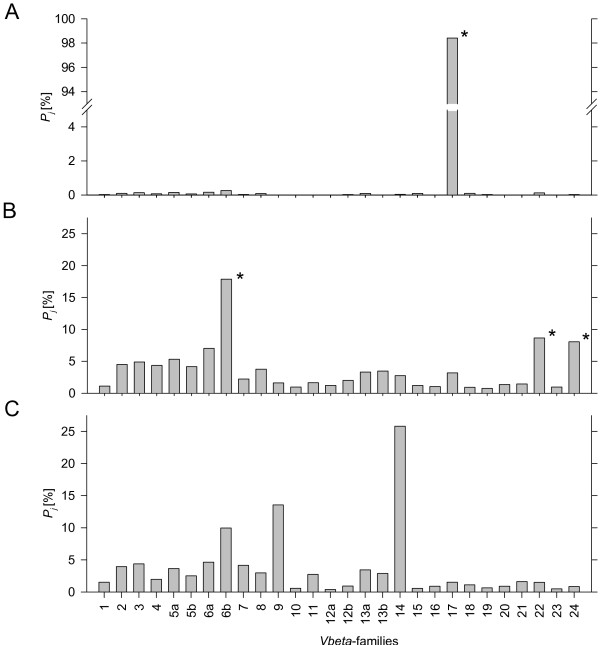
**Relative quantification of TCR-Vβ-families: Examples of clinical samples**. (A) T prolymphocytic leukemia, (B) suspected T/NK-LGL lymphoproliferation, (C) Colon carcinoma tissue. Peripheral blood *Vβ*-chain values (A, B) exceeding mean plus two standard deviations in healthy controls are marked with asterisk. *P*_*j*_: *Vβ*-chain relative expression.

The method was applied to blood samples of patients with hematological diseases and to colon carcinoma tissues. Three examples with representative *Vβ *patterns were shown in Figure 4. Pattern *A *showed the T-cell family repertoire of a patient affected by T prolymphocytic leukemia with 98.0 leukocytes per nl and 89% CD4/CD8 positive cells (FACS analysis, data not shown) in peripheral blood. The pathological clone expressed *Vβ*-family 17 and represented more than 98% of all TCR *α*/*β *positive lymphocytes in peripheral blood. Pattern *B *showed a patient with suspected T-/NK-LGL lymphoproliferation. *Vβ*-families 6b, 22, and 24 exceeded mean plus two standard deviations as determined in twenty healthy controls. Therefore, a monoclonal TCR α/β expressing lymphoproliferation was unlikely and findings had to be interpreted as reactive T cell expansion. Pattern *C *showed the *Vβ *family distribution in a representative colon carcinoma tissue sample. A higher percentage of *Vβ*-family 14 compared to other families was observed. If TCR restriction in the tumor compartment reflects a spontaneous expansion of tumor-specific T-cell clones is still matter of debate.

Compared to techniques characterizing the CDR3 region (e.g. spectrotyping, SSCP, DGGE), the method described is less time-consuming and useful for high-throughput screening analyses. As for spectrotyping, conclusive proof of clonality can only be achieved by sequencing. Approaches like TC landscape combine CDR3 length analysis with *Vβ*-family quantification by FACS quantification in order to improve sensitivity [5]. Due to higher number of detectable families and higher sensitivity, the use of a high-throughput PCR-based quantification algorithm could make a combined approach even more effective [16].

## Conclusion

We present a novel and versatile approach for high-throughput *Vβ *family quantification. Our approach is suitable for samples from blood or bone marrow as well as from tissue. Because of high reproducibility, comparative analyses of samples in different time points or from different compartments are possible.

## Competing interests

The authors declare that they have no competing interests.

## Authors' contributions

SO and AF carried out most of the molecular applications. SO developed the mathematical algorithm and did the calculations and the data analysis. AB did the qRT PCR quantification of HAC and PBGD. DN was responsible for collection and storage of blood and tissue samples. DS and JB built up qRT PCRs for family quantification which were used as basis for the LightCycler approach. ET and UK coordinated the laboratory work and helped to draft the manuscript. All authors read and approved the final manuscript.
